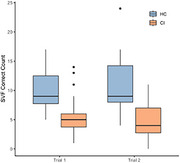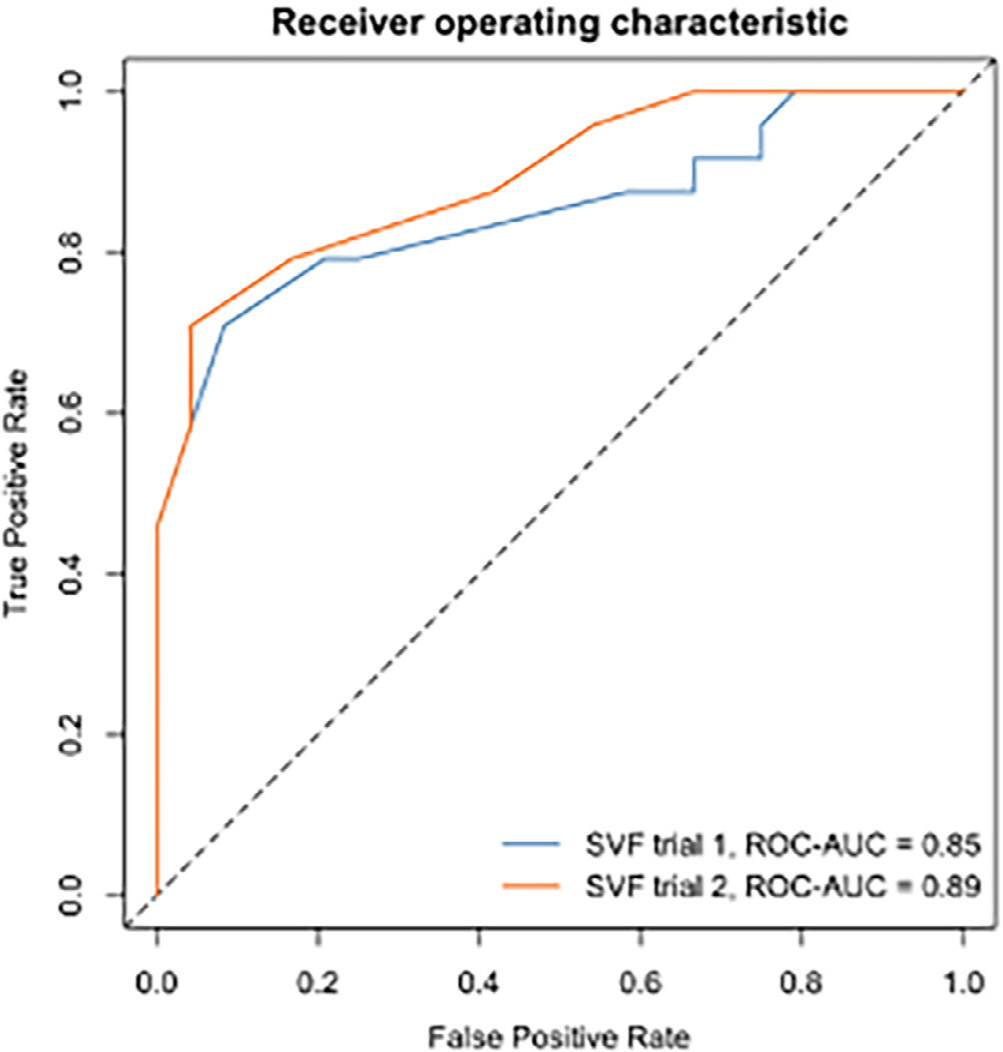# Digital semantic fluency analysis detects dementia in multilingual Kenyan adults

**DOI:** 10.1002/alz70856_103811

**Published:** 2025-12-25

**Authors:** Karen Blackmon, Levi A. Muyela, Johannes Tröger, Elisa Mallick, Nicklas Linz, Alexandra König, Anne Njoki Gitere, Anne Nyambura Njogu, Irene Meier, Vaibhav Narayan, Zul Merali, Chinedu Udeh‐Momoh

**Affiliations:** ^1^ Geisel School of Medicine at Dartmouth, Hanover, NH, USA; ^2^ Dartmouth Hitchcock Medical Center, Lebanon, NH, USA; ^3^ Brain and Mind Institute, Aga Khan University, Nairobi, Kenya; ^4^ ki:elements GmbH, Saarbrücken, Germany; ^5^ Davos Alzheimer's Collaborative, Wayne, PA, USA; ^6^ Aga Khan University, The Brain and Mind Institute, Nairobi, Nairobi, Kenya; ^7^ Global Brain Health Institute, University of California, San Francisco, NC, USA; ^8^ FINGERS Brain Health Institute, Solna, Stockholm, Sweden; ^9^ Wake Forest University, School of Medicine, Winston‐Salem, NC, USA

## Abstract

**Background:**

Dementia is a growing health challenge in Africa, where multilingualism and low literacy complicate diagnosis. Because most cognitive assessment tools do not include African populations in their development or validation, their applicability is limited. The semantic verbal fluency (SVF) task is promising due to its adaptability to multiple languages and low literacy levels. When administered twice, SVF can reveal practice effects that are typically reduced in dementia, offering additional diagnostic value. This study examines how well a digital, tablet‐based SVF, scored by an automated system, discriminates older Kenyan adults with and without dementia.

**Method:**

Data were collected as part of a Davos Alzheimer's Collaborative‐funded study in Nairobi, Kenya. We recruited 24 cognitively unimpaired controls (mean age=65.42; 12 females) and 24 education‐ and age‐matched participants with clinically diagnosed dementia (mean age=71.13; 7 females). All spoke a mix of three languages and had diverse educational backgrounds. They completed a neuropsychological battery including two consecutive 60‐second SVF tasks (animal naming), allowing language‐switching. We employed ki:elements’ SIGMA speech analysis pipeline to identify correct responses and generate traditional metrics (e.g., word count) plus novel features (pauses, speech rate, clustering, word frequency). Non‐parametric comparisons and logistic regression models were used to evaluate group differences and classification accuracy on each SVF trial

**Result:**

Groups differed significantly in word count on both the first (χ^2^=17.60, *p* <.001, η^2^=.36) and second (χ^2^=22.12, *p* <.001, η^2^=.46) trials, with a greater difference in the second trial. Performance change across trials (χ^2^=4.77, *p* <.05, η^2^=.08) indicated that repeated administration widened the gap, potentially reflecting practice effects in controls and reduced improvement in dementia. Logistic regression showed high discriminative power (ROC‐AUC=.85 in trial 1, .89 in trial 2).

**Conclusion:**

These findings demonstrate that an automatically scored, multilingual SVF test can reliably detect dementia in older Kenyan adults, particularly when practice effects are considered. Digital SVF holds promise as a culturally adaptable, rapid, and sensitive tool for cognitive screening in low‐resource, multilingual settings. By accommodating linguistic diversity, it could increase access to earlier diagnosis and foster more inclusive clinical research in Africa. This approach highlights the potential for bridging gaps in equitable dementia care by delivering cost‐effective and scalable solutions.